# Consumption of Barley, Buckwheat and Quinoa in the United States: Associations with Diet and Metabolic Health

**DOI:** 10.3390/nu17142335

**Published:** 2025-07-17

**Authors:** Namrata Sanjeevi, Sarah Elizabeth Stella, Pablo Monsivais

**Affiliations:** Department of Nutrition and Exercise Physiology, Elson S. Floyd College of Medicine, Washington State University, Spokane, WA 99202, USA; namrata.sanjeevi@wsu.edu (N.S.);

**Keywords:** barley, buckwheat, quinoa, diet quality, metabolic health

## Abstract

**Background/Objectives**: Despite the health benefits of fiber intake, most Americans do not meet the dietary recommendations for this nutrient. With barley, buckwheat and quinoa containing several nutrients, including fiber, the integration of these foods into the American diet could improve diet quality and health. The purpose of this study is to examine the consumption patterns of barley, buckwheat and quinoa and its association with diet and metabolic health markers. **Methods:** We used National Health and Nutrition Examination Survey (NHANES) 2013–2018 data. Adults who had consumed barley, buckwheat and quinoa, as determined by food codes representing these foods, in at least one of the two 24 h recalls were categorized as consumers. Due to the very low prevalence of buckwheat consumption, the associations of consumption with diet and health markers were examined only for barley and quinoa. **Results:** The prevalence of barley, buckwheat and quinoa consumption among US adults were 0.36%, 0.04% and 1.07%, respectively. Compared with non-consumers, barley and quinoa consumers had significantly greater diet quality and higher intakes of potassium and fiber. While barley consumption was associated with a lower body mass index, waist circumference and glycohemoglobin, quinoa consumption was associated with a lower body mass index, waist circumference, triglycerides and total cholesterol. **Conclusions:** In this study, we found an overall low prevalence of the consumption of barley, buckwheat and quinoa among US adults. The consumers of barley and quinoa had better dietary intake compared with non-consumers. Although better metabolic health in consumers may be explained by their overall healthier dietary patterns, understanding the barriers and facilitators to the intake of these foods could inform efforts to improve diet quality and health.

## 1. Introduction

Unhealthy dietary patterns are a significant contributor to the prevalence of chronic diseases in the United States (US) [1,2,3,4]. Despite the evidence supporting the role of dietary fiber in the promotion of optimal metabolic health, about 90% of the US population is estimated to fall short of meeting the recommended intake for this nutrient [5]. The microbial fermentation of dietary fiber into short chain fatty acids has been shown to regulate glucose and lipid metabolism [6]. Furthermore, the intake of dietary fiber has been associated with a reduction in the risk of cardiovascular disease (CVD) via mechanisms such as the regulation of lipids, body weight, glucose metabolism, blood pressure and inflammation [7]. Promoting the consumption of foods rich in dietary fiber could help mitigate the burden of noncommunicable disease in the US.

The inclusion of whole grain products could facilitate the consumption of dietary fiber of all varieties; however, 98% of the US population do not meet the recommended intake for whole grains [8,9,10]. Given that barley, buckwheat and quinoa contain more fiber than refined grain products as well as certain other whole grains [11], the incorporation of these foods into the diet could be an effective way to increase dietary fiber in the US population.

Barley is a cereal grain mostly used for livestock feed and malting, with only a small portion being consumed by humans in the pearl form or as flour, despite being high in fiber and including moderate amounts of protein, calcium, phosphorous and B vitamins [12]. Buckwheat is a gluten-free, pseudocereal with a wide range of beneficial effects, potentially due to its bioactive components [13]. Quinoa is a pseudo-grain that shares some of the benefits of barley and buckwheat, while also being a rich source of antioxidants [14,15,16]. Barley, buckwheat and quinoa are known to contain high levels of dietary fiber and randomized controlled trials provide support for the beneficial effects of barley, buckwheat and quinoa consumption on CVD markers [17,18,19,20]. However, socioeconomic and structural barriers could hinder the availability, accessibility and affordability of these foods [21,22]. An understanding of the sociodemographic characteristics of consumers, the associations of consumption with metabolic health in free-living individuals, as well as the monetary costs of these foods is currently lacking.

The purpose of this study is to characterize the sociodemographic, dietary and metabolic profiles associated with the consumption of barley, buckwheat and quinoa in the US population. In addition, we examined the eating context and retail sources of these three foods, as well as their retail monetary cost, in comparison with rice.

## 2. Materials and Methods

### 2.1. Data Sources

The NHANES is a series of nationally representative, cross-sectional surveys that assesses the nutrition and health status of the noninstitutionalized civilian U.S. population [23]. We utilized 2-year NHANES cycles from 2013 to 2014 through 2017 to 2018. To obtain the cost estimates of barley, buckwheat, quinoa and rice, we used the Purchase to Plate National Average Price (PP-NAP) 2017–2018, which provides price estimates for foods reported in the dietary component of NHANES. The PP-NAP provides food prices (in US Dollars) that are the average of price data collected from grocery stores. More details on PP-NAP are provided elsewhere [24].

### 2.2. Study Population

Analyses examining demographic characteristics and dietary intake included adults, aged 20 years or older (n = 14,806). Analyses involving body mass index (BMI), waist circumference, glycohemoglobin (HbA1c) and total cholesterol included non-pregnant adults, aged 20 years or older (n = 14,633), whereas analyses involving other metabolic health markers included a fasting subsample of non-pregnant participants, aged 20 years or older (n = 6397). Analyses examining individual foods and sources of acquisition included only consumers of barley, buckwheat and quinoa, aged 3 years or older (n = 116 for barley; n = 17 for buckwheat; n = 192 for quinoa). Due to the use of publicly available, de-identified datasets, the current study was considered ‘not human subjects research’ and did not require IRB approval. The data collection protocol for NHANES has been approved by the National Center for Health Statistics’ Research Ethics Review Board [25]. Written informed consent was obtained from the participants, with parents or guardians providing permission for participation among children aged less than 18 years [26].

### 2.3. Identifying Consumers of Barley, Buckwheat and Quinoa

NHANES uses up to two 24 h dietary recalls [27] to collect dietary information from participants; for participants who completed only one 24 h recall, dietary data from one recall was used. The 24 h recalls are designed to collect detailed information on all foods and beverages consumed in the past 24 h, including estimates of the amount consumed [27]. Trained interviewers use the USDA Automated Multiple Pass Method for conducting the 24 h dietary recall. The first 24 h dietary recall is conducted in the Mobile Examination Center (MEC) and the second recall is completed by telephone 3–10 days later. Dietary intakes are self-reported by participants aged ≥12 years. For children aged 6–11 years, proxy-assisted dietary interviews are conducted, whereas 24 h recalls are collected from proxies for children aged <6 years [28].

Publicly available individual food files [29,30] and food code description files [31] were used to identify consumers of barley, buckwheat and quinoa. Food codes corresponding to barley, buckwheat and quinoa foods were identified from the food code description and ingredient list. Only two foods containing barley (i.e., oxtail soup and Puerto Rican bread) and one food containing buckwheat (i.e., injera bread) were identified based on the ingredient list. Since the barley and buckwheat content in these foods is unclear, food codes representing these foods were not used in the classification of individuals as consumers. Participants with an occurrence of food codes representing barley, buckwheat and quinoa in at least one of the two 24 h recalls were classified as consumers. All others were categorized as non-consumers. In addition, among consumers, the description of the individual barley, buckwheat and quinoa foods consumed were determined using food code description information. The source of acquisition (i.e., retail and other sources) of these foods was obtained from the individual food files.

### 2.4. Dietary Intake

Dietary intake variables of interest include diet quality and nutrient intake. For participants who completed two 24 h recalls, the average of the recall data was used [32,33]. When only one 24 h recall was available, data from one recall was used.

Overall dietary quality was assessed using the Healthy Eating Index-2015 (HEI-2015), a measure that evaluates conformance of dietary intake to the 2015–2020 Dietary Guidelines for Americans (DGA) [34]. The HEI-2015 consists of nine adequacy components (i.e., total fruits, whole fruits, total vegetables, greens and beans, whole grains, dairy, total protein foods, seafood and plant proteins, and fatty acids) and four moderation components (refined grains, sodium, added sugars and saturated fat). Greater intakes of adequacy components receive higher scores, whereas moderation components are scored so that higher scores are assigned to lower intakes. Thus, higher scores in each HEI-2015 component are reflective of a greater alignment of intake with recommendations. The total HEI-2015 score is a summation of the scores for the adequacy and moderation components.

We estimated the intakes of nutrients of public health concern [35] (i.e., potassium (mg), iron (mg), calcium (mg), vitamin D (mcg) and fiber (gm)) from the NHANES data release. Fiber density was then calculated as grams per 1000 kilocalories (kcal).

### 2.5. Cost Estimates of Barley, Buckwheat, Quinoa and Rice

The price for 100 g of barley, buckwheat, quinoa, white rice and brown rice were obtained from the PP-NAP 2017–2018. Based on the ounce equivalents and kcal present in 100 g, we calculated the cost per ounce equivalent and cost per 100 kcal for these foods. The type of price measure could affect the understanding of which foods are more expensive [36]; thus, cost per ounce equivalent and cost per 100 kcal were both utilized in this study.

### 2.6. Sociodemographic and Dietary Characteristics

Sociodemographic characteristics included for the analyses were participants’ age, sex, race/ethnicity (Mexican American/Other Hispanic; non-Hispanic White; non-Hispanic Black; non-Hispanic Asian/other), education level (high school or less; some college/associate degree; college graduate or above) and family income-to-poverty-ratio.

### 2.7. Metabolic Health Markers

The following metabolic health markers were considered: BMI, waist circumference, HbA1c, fasting plasma glucose (FPG), total cholesterol, triglycerides and low-density lipoprotein cholesterol (LDL-C). BMI, calculated from measured height and weight, and waist circumference were obtained from participants attending the MEC. Blood specimens drawn from participants at the MEC were used to measure HbA1c [37] and total cholesterol [38]. Furthermore, a fasting subsample of participants attending the morning examination session were assessed for FPG [39] and triglycerides [40]. Due to changes in laboratory instrumentation, FPG values from NHANES 2015–2016 and 2017–2018 were matched to NHANES 2013–2014 using backward Deming’s regression [39,41,42]. LDL-C was estimated using the Friedewald equation, using total cholesterol, triglycerides and high-density lipoprotein cholesterol values [40].

### 2.8. Statistical Analyses

Since the number of buckwheat consumers in NHANES 2013–2018 was nine, we did not examine the demographic, dietary and metabolic profiles associated with buckwheat consumption. Linear regression and chi-square tests examined the differences between consumers and non-consumers of barley and quinoa for continuous and categorical demographic characteristics, respectively. Linear regression examined the differences in dietary intake between consumers and non-consumers of barley and quinoa. Linear regression analyses examined the associations of barley and quinoa consumption with metabolic health markers in non-pregnant US adults. Analyses using BMI and waist circumference as outcomes were adjusted for age, sex, race/ethnicity, education level and income to poverty ratio, whereas analyses using HbA1c, FPG, LDL-C, total cholesterol and triglycerides as outcomes were adjusted for age, sex, race/ethnicity, education level, income-to-poverty-ratio as well as BMI. Analyses accounted for the complex survey design of NHANES, and appropriate sample weights were used.

Among consumers, frequency tables were used to obtain the distribution of individual foods, and sources of acquisition of barley, buckwheat and quinoa. All analyses were conducted in the SAS (version 9.4; SAS Institute, Inc., Cary, NC, USA) software.

## 3. Results

### 3.1. Demographic Characteristics of Consumers and Non-Consumers of Barley and Quinoa

Table 1 indicates the differences in demographic characteristics between consumers and non-consumers of barley and quinoa. The proportion of barley consumers who were non-Hispanic Asians/other race and with a college degree or above was greater than the proportion of non-consumers with the corresponding race/ethnicity and education level. Barley consumption did not significantly differ by age, sex and income to poverty ratio. In contrast, quinoa consumption was more common in females compared with males and quinoa consumers had a greater income-to-poverty-ratio. The consumption of quinoa also significantly differed by race/ethnicity and education level. The proportion of quinoa consumers who were non-Hispanic white and with a college degree or above was greater than the proportion of non-consumers with the corresponding race/ethnicity and education level. For both barley and quinoa, the proportion of consumers classified as non-Hispanic black was lower than other race/ethnicity categories.

### 3.2. Dietary Intake of Consumers and Non-Consumers of Barley and Quinoa

Compared with non-consumers, barley and quinoa consumers had a significantly greater diet quality, as indicated by HEI-2015 total score (Table 2). Barley consumers also had significantly higher scores for all HEI-2015 components, except for greens and beans, total protein, fatty acids and refined grains. Quinoa consumers had significantly higher scores for all dietary components, except for total dairy and sodium. Significant differences in nutrient intake also were observed, such that barley consumers had significantly higher intakes of potassium, iron and fiber than non-consumers. Compared with quinoa non-consumers, consumers had significantly higher intakes of potassium and fiber.

### 3.3. Association of Barley and Quinoa Consumption with Metabolic Health Markers

Table 3 indicates the association of barley and quinoa consumption with metabolic health markers. While barley consumption was significantly associated with a lower BMI, waist circumference and HbA1c, its associations with FPG, LDL-C, total cholesterol and triglycerides were not statistically significant. Quinoa consumption was significantly related to a lower BMI, waist circumference, total cholesterol and triglycerides; however, its associations with other metabolic health markers were not significant.

### 3.4. Eating Context and Sources of Barley, Buckwheat and Quinoa

Table 4 indicates the distribution of individual foods and dishes containing barley, buckwheat, or quinoa reported by consumers. Barley soup, quinoa (without any fat) and buckwheat pancakes were the most commonly consumed foods within each of the three food categories. Table 5 indicates the distribution of retail and other sources of barley, buckwheat and quinoa acquisition. Consumers of barley, buckwheat and quinoa predominantly obtained these foods from grocery stores/supermarkets. Restaurants/fast food joints were the second most common source for these foods, whereas convenience stores represented just about 1% of the sources of barley, buckwheat and quinoa.

### 3.5. Cost of Barley, Buckwheat, Quinoa, Brown Rice and White Rice

Table 6 indicates that the cost estimates for quinoa and buckwheat were higher than that for barley, brown rice and white rice. Although the cost for barley was lower than that of brown rice, it was greater than the cost estimates for white rice.

## 4. Discussion

In this study, we found that barley, buckwheat and quinoa were sparsely consumed by US adults, with prevalences of 0.36%, 0.04% and 1.07%, respectively. The slightly higher prevalence of quinoa consumption may be attributed to the expansion of quinoa production, supply and popularity in recent years [43,44,45]. Significant differences in demographic characteristics and dietary intake were observed by consumption status, such that barley and quinoa consumers had a greater education level and diet quality compared with non-consumers. The consumption of barley and quinoa was also significantly associated with better metabolic health markers. While barley and quinoa could have independently contributed to improved diet quality and health markers in consumers, it is also possible that the consumers of these foods have an overall healthful diet and thus better metabolic health than non-consumers. Nevertheless, barley, buckwheat and quinoa are rich sources of fiber and several other nutrients [12,13,14,15], and consumers of barley and quinoa also showed significantly greater intakes of total dietary fiber. Taken together, these findings suggest that efforts to increase the consumption of these foods could be vital to improve diets and promote the health of Americans.

Compared with barley and quinoa non-consumers, a significantly greater proportion of consumers had an education level of college degree or above. Differences were also observed by race/ethnicity, such that a significantly greater proportion of barley and quinoa consumers were non-Hispanic Asian/other and non-Hispanic White, respectively. Although quinoa consumers had a higher income-to-poverty ratio than non-consumers, the income-to-poverty ratio did not significantly differ between barley consumers and non-consumers. The demographic pattern of quinoa consumption is consistent with previous research indicating greater whole grain purchasing [46] and consumption [47] among individuals of higher socioeconomic status and who were non-Hispanic White. The lack of significant difference in income-to-poverty ratio by barley consumption status may be attributed to the greater affordability of barley compared with other whole grain foods. Consistently, in this study, we found that the cost of barley was lower than buckwheat, quinoa and brown rice, and was only slightly higher than white rice. The barley consumption pattern observed among individuals from non-Hispanic Asian/other race/ethnicity may be attributed to the integration of barley as a staple food in Asian culture [48]. For both barley and quinoa, the proportion of consumers who were non-Hispanic black was lower than other race/ethnicity categories. Taken together with previous research suggesting disparities in whole grain and fiber intake among non-Hispanic Black adults [47,49], future studies could investigate the potential of increasing fiber intake in this racial/ethnic group via the incorporation of barley, buckwheat and quinoa.

The average HEI-2015 score for barley and quinoa consumers was greater than that of non-consumers of these foods. Compared with barley and quinoa non-consumers, consumers also scored higher in several HEI-2015 dietary components, such as total fruits, whole fruits, total vegetables, whole grains, seafood and plant protein and added sugar. The overall healthful intake among consumers could be attributed to sociodemographic factors, such as a greater education level, which is known to be associated with greater nutrition knowledge and improved food purchasing behaviors [50]. Furthermore, the common inclusion of barley and quinoa in soups/salads is expected to concomitantly increase the intake of vegetables, fruits and plant proteins in the diet [51,52]. We also observed that consumers had greater intakes of some shortfall nutrients, including potassium and fiber, possibly due to the healthfulness of dietary intake among barley and quinoa consumers.

In the current study, barley and quinoa consumption was significantly associated with better metabolic health markers. Specifically, barley and quinoa consumption was related to a lower BMI and waist circumference, even after adjustment for demographic characteristics. After additionally adjusting for BMI, barley consumption was associated with lower HbA1c, whereas quinoa consumption was associated with lower total cholesterol and triglycerides. These associations remained statistically significant even after adjustment for overall diet quality. For example, even after adjustment for overall diet quality, quinoa consumption was significantly associated with lower triglycerides (estimate± standard error = −15.16 ± 7.24; *p*-value= 0.04). Furthermore, the strength of these associations implies that the study findings are clinically meaningful. Although these findings may be explained by the overall healthier dietary patterns in consumers, they are somewhat consistent to randomized controlled trials indicating that barley and quinoa supplementation could reduce cardiovascular risk factors in adults [17,18].

We found that grocery stores/supermarkets constituted the major retail source for acquisition of barley, buckwheat and quinoa; a finding that is similar to our previous analysis examining the source of acquisition of lentils and dried peas [53]. While this result could be explained by the greater use of grocery stores/supermarkets for food purchasing among Americans [54], it could also be reflective of the reduced availability of healthful foods in convenience stores [55]. Furthermore, the greater neighborhood availability of convenience stores has been associated with lower whole grain consumption in young adults [56]. With the higher density of convenience stores in low-income neighborhoods [57], the increased availability of whole grains and plant proteins in these stores could represent an opportunity to improve the accessibility of these foods among low-income households.

The limitations of this study include the use of a cross-sectional design, which limits the inferences on causality. Possible residual confounding could have overestimated some of the study associations. Self-reported dietary intake obtained from 24 h dietary recalls could be subject to misreporting due to social desirability or recall bias [58,59] and may not be reflective of long-term intake. Furthermore, the dietary recall data does not distinguish between pearl and hulled barley, which differ in whole grain content and other nutrition properties [60]. The lack of distinction between pearl and hulled barley could have underestimated the associations of barley consumption with metabolic health markers. The low prevalence of buckwheat consumption precluded the investigation of sociodemographic and dietary differences between consumers and non-consumers. Nevertheless, the study findings are strengthened by using nationally representative data and employing two 24 h recalls, when available, for the estimations of dietary intake. To our knowledge, this is the first study to comprehensively examine the consumption patterns of barley, buckwheat and quinoa, its associations with overall dietary intake and metabolic health as well as the eating context and monetary costs of these foods.

## 5. Conclusions

In this nationally representative sample of US adults, we found an overall low prevalence of the consumption of barley, buckwheat and quinoa. Sociodemographic differences were observed between consumers and non-consumers of barley and quinoa, such that a significantly greater proportion of consumers had an education level of college degree or above. The consumers of barley and quinoa had better diet quality and metabolic health markers than non-consumers. The cost estimates of quinoa and buckwheat were higher than that of barley. Future research investigating the barriers, such as cost and availability, to barley, buckwheat and quinoa consumption could inform efforts to increase the intake of these foods.

## Figures and Tables

**Table 1:** Demographic characteristics of US adults by consumption of barley and quinoa ^a^: NHANES 2013–2018.

	Consumption of Barley		Consumption of Quinoa	
Characteristics ^b^	No	Yes	No	Yes
Adults (aged 20 years or older) Age	n = 14,748 47.5 ± 0.4	n = 58 54.6 ± 3.7	n = 14,683 47.5 ± 0.4	n = 123 44.9 ± 2.6
Sex				
Male	48.7	50.1	49 ***	25
Female	51.3	49.9	51	75
Race/ethnicity				
Mexican American/Other Hispanic	15.7 ***	3.1	15.7 **	9.9
Non-Hispanic White	63.4	57.2	63.3	75.6
Non-Hispanic Black	11.2	2.1	11.3	2
Non-Hispanic Asian/Other	9.6	37.6	9.7	12.5
Education level				
High school or less	36.5 ***	18.3	36.8 ***	1.8
Some college/associate degree	32.6	18.4	32.5	31
College graduate or above	30.9	63.3	30.7	67.2
Income-to-poverty-ratio	2.98 ± 0.05	3.27 ± 0.39	2.97 ± 0.05 ***	3.97 ± 0.17

NHANES, National Health and Nutrition Examination Survey.** *p* < 0.01, *** *p* < 0.001, ^a^ Days 1 and 2 of 24 h dietary recall were used for participants who completed both days of diet recall; day 1 diet recall was used for other participants, ^b^ Data are represented as mean ± standard error of mean or %.

**Table 2:** Dietary intake of US adults by consumption of barley and quinoa ^a^: NHANES 2013–2018.

	Consumption of Barley		Consumption of Quinoa	
Characteristics ^b^	No	Yes	No	Yes
Adults (aged 20 years or older)	n = 14,748	n = 58	n = 14,683	n = 123
Healthy Eating Index 2015 total score	53.1 ± 0.37 ***	62.3 ± 2.22	53 ± 0.36 ***	70.2 ± 1.32
HEI-2015 component scores				
Total fruits	2.2 ± 0.04 ***	3.7 ± 0.29	2.2 ± 0.04 ***	3.6 ± 0.23
Whole fruits	2.4 ± 0.05 ***	4.1 ± 0.28	2.4 ± 0.05 ***	4.0 ± 0.20
Total vegetables	3.2 ± 0.03 ***	4.3 ± 0.20	3.2 ± 0.03 ***	4.3 ± 0.16
Greens and beans	2.0 ± 0.04	2.6 ± 0.55	2.0 ± 0.04 ***	3.7 ± 0.30
Whole grains	2.8 ± 0.06 ***	4.7 ± 0.54	2.8 ± 0.06 ***	6.2 ± 0.35
Total dairy	5.2 ± 0.06 **	3.8 ± 0.47	5.2 ± 0.06	4.6 ± 0.46
Total protein	4.5 ± 0.02	4.5 ± 0.20	4.5 ± 0.02 ***	4.7 ± 0.05
Seafood and plant protein	2.9 ± 0.04 **	3.9 ± 0.33	2.9 ± 0.04 ***	4.3 ± 0.13
Fatty acid ratio	4.9 ± 0.05	6.1 ± 0.62	4.9 ± 0.05 ***	7.1 ± 0.36
Sodium	4.1 ± 0.06 ***	2.4 ± 0.38	4.1 ± 0.06	3.9 ± 0.36
Saturated fat	5.6 ± 0.06 ***	7.6 ± 0.55	5.6 ± 0.06 ***	6.9 ± 0.33
Added sugar	7.0 ± 0.07 ***	9.0 ± 0.27	7.0 ± 0.07 ***	9.1 ± 0.16
Refined grain	6.3 ± 0.06	5.7 ± 0.61	6.3 ± 0.06 ***	8.0 ± 0.33
Nutrient intake				
Potassium, mg	2608.73 ± 21.2 **	3361.81 ± 222.13	2605.78 ± 21.5 ***	3134.33 ± 145.71
Iron, mg	14.3 ± 0.11 ***	18.1 ± 0.76	14.2 ± 0.11	16.4 ± 1.15
Calcium, mg	954.77 ± 9.10	962.55 ± 41.67	954.67 ± 8.98	966.26 ± 65.09
Vitamin D, mcg	4.6 ± 0.07	4.9 ± 0.5	4.6 ± 0.07	5.2 ± 0.5
Fiber, g per1000 kcal	8.4 ± 0.07 ***	11.9 ± 0.67	8.4 ± 0.07 ***	12.9 ± 0.51

NHANES, National Health and Nutrition Examination Survey, ** *p* < 0.01, *** *p* < 0.001, ^a^ Days 1 and 2 of 24 h dietary recall were used for participants who completed both days of diet recall; day 1 diet recall was used for other participants, ^b^ Data are represented as mean ± standard error of mean or %.

**Table 3:** Association ^a^ of barley and quinoa consumption with metabolic health markers in US adults ^b^, aged 20 years or older, NHANES 2013–2018.

	BMI	WC	HbA1c	FPG	LDL-C	Total Cholesterol	Triglycerides
	Estimate ± Standard Error						
Barley							
Non-consumers (reference category)	--	--	--	--	--	--	--
Consumers	−2.62 ± 0.82 **	−6.43 ± 2.15 **	−0.14 ± 0.06 *	−4.70 ± 2.76	−5.55 ± 7.99	−13.29 ± 7.87	5.09 ± 26.33
Quinoa							
Non-consumers (reference category)	--	--	--	--	--	--	--
Consumers	−3.29 ± 0.56 ***	−9.13 ± 1.14 ***	−0.03 ± 0.07	2.30 ± 3.63	−5.97 ± 4.50	−6.58 ± 3.03 *	−19.19 ± 6.35 **

* *p* < 0.05, ** *p* < 0.01, *** *p* < 0.001. BMI, body mass index; WC, waist circumference; HbA1c, glycohemoglobin; FPG, fasting plasma glucose; LDL-C, low-density lipoprotein cholesterol. ^a^ Associations of consumption with BMI and WC were adjusted for age, sex, race/ethnicity, education level and income-to-poverty-ratio; associations with all other metabolic health markers were additionally adjusted for BMI. ^b^ Pregnant women were excluded from the sample.

**Table 4:** Distribution of individual foods and dishes containing barley, buckwheat or quinoa, reported by consumers ^a^, NHANES 2013–2018.

Individual Foods and Dishes	Proportion of Occurrence (%)
Barley ^b^	
Barley soup, home recipe, canned, or ready-to-serve	55.3
Barley, no added fat	17.1
Barley soup, sweet, with or without nuts, Asian Style	11.8
Barley, NS as to fat	7.9
Barley, cooked, fat not added/NS as to fat added in cooking	5.3
Bread, barley	1.3
Bread, barley, toasted	1.3
Quinoa	
Quinoa, no added fat	31.6
Quinoa, NS as to fat	22.2
Quinoa, fat added	14.6
Quinoa, cooked, no fat added	15.2
Quinoa, cooked, NS as to fat	10.5
Quinoa, cooked, fat added	5.9
Buckwheat	
Pancakes, buckwheat	50
Buckwheat groats, fat not added in cooking	21.4
Buckwheat groats, NS as to fat	14.3
Buckwheat groats, cooked, fat added in cooking	7.1
Buckwheat groats, fat added in cooking	7.1

NHANES, National Health and Nutrition Examination Survey; NS, not specified. ^a^ Includes consumers aged 3 years or older. ^b^ The food, ‘barley cereal, baby food’ was excluded from analyses.

**Table 5:** Distribution of retail and other sources of barley, buckwheat and quinoa among consumers ^a^, NHANES 2013–2018.

Retail and Other Acquisition Sources	Proportion of Occurrence (%)
Barley, buckwheat and quinoa	
Grocery store/supermarket	86.1
Restaurant/fast food joint	5.2
From someone else/gift	3.8
Mail order purchase	2.1
Convenience store	1.1
Cafeteria not in a K-12 school	1.1
Child/adult home care	0.4
Community food program	0.4

NHANES, National Health and Nutrition Examination Survey. ^a^ Includes consumers aged 3 years or older.

**Table 6:** Cost, in US Dollars, of barley, buckwheat, quinoa, brown rice and white rice, based on Purchase to Plate National Average Prices 2017–2018.

Food	Price per 100 Grams	Cost per oz eq ^a^	Cost per 100 kcal ^a^
Barley, no added fat	0.1085	0.0897	0.0877
Buckwheat groats, no added fat	0.3561	0.2950	0.3864
Quinoa, no added fat	0.3934	0.3251	0.3278
Brown rice, NS as to fat ^b^	0.2312	0.1904	0.1888
White rice, no added fat	0.0896	0.0734	0.0694

NS, not specified; oz eq, ounce equivalents. ^a^ Calculated using information on ‘Buckwheat groats, NS as to fat’ from the individual food files. ^b^ ‘Brown rice, NS as to fat’ is presented in the place of ‘Brown rice, no added fat’ as the price was more comparable to other brown rice products.

## Data Availability

The datasets used for the study analysis were obtained from https://wwwn.cdc.gov/nchs/nhanes/default.aspx. Accessed on 20 November 2022.

## Abbreviations

The following abbreviations are used in this manuscript:

NHANES	National Health and Nutrition Examination Survey
PP-NAP	Purchase to Plate National Average Price
BMI	Body mass index
HbA1c	Glycohemoglobin
FPG	Fasting plasma glucose
LDL-C	Low-density lipoprotein cholesterol
MEC	Mobile examination center
HEI-2015	Healthy Eating Index 2015
